# Trace Element and Mineral Levels in Serum, Hair, and Urine of Obese Women in Relation to Body Composition, Blood Pressure, Lipid Profile, and Insulin Resistance

**DOI:** 10.3390/biom11050689

**Published:** 2021-05-04

**Authors:** Alexey A. Tinkov, Paweł Bogdański, Damian Skrypnik, Katarzyna Skrypnik, Anatoly V. Skalny, Jan Aaseth, Margarita G. Skalnaya, Joanna Suliburska

**Affiliations:** 1 Laboratory of Ecobiomonitoring and Quality Control, Yaroslavl State University, Sovetskaya St., 14, 150000 Yaroslavl, Russia; tinkov.a.a@gmail.com (A.A.T.); skalny3@microelements.ru (A.V.S.); skalnaya@yandex.ru (M.G.S.); 2 Department of Treatment of Obesity, Metabolic Disorders and Clinical Dietetics, Poznań University of Medical Sciences, Szamarzewskiego St. 84, 60-569 Poznań, Poland; pbogdanski@ump.edu.pl (P.B.); damian.skrypnik@gmail.com (D.S.); 3 Department of Human Nutrition and Dietetics, Poznan University of Life Science, ul. Wojska Polskiego 31, 60-624 Poznan, Poland; katarzyna.skrypnik@up.poznan.pl; 4 Department of Research, Innlandet Hospital Trust, 2380 Brumunddal, Norway; jaol-aas@online.no

**Keywords:** obesity, metabolic syndrome, insulin resistance, copper, selenium

## Abstract

The objective of this study was to evaluate serum, hair, and urinary trace element and mineral content in normal-weight and obese women in relation to metabolic risk factors. A total of 80 women aged 30–70 y.o. were enrolled in the obese group (n = 40) and normal-weight group (n = 40). Serum, hair, and urinary trace element and mineral levels were assessed using inductively coupled plasma spectrometry. Body fat percentage was evaluated using bioimpedance. Obese subjects were characterized by significantly higher body fat percentage, blood pressure, serum triglyceride concentration, and insulin resistance. Serum Ca, Fe, Mg, Se, V, Zn levels, hair Fe, Mg, V content, and urinary Se and V concentrations were found to be lower in obese subjects as compared to lean controls. In turn, serum Cu and urinary Fe levels in obese women were characterized by a significant increase. In multiple regression models serum Cu, Se, and Zn levels were significantly associated with BMI even after adjustment for blood biochemistry, body composition, and blood pressure. Serum trace element and mineral levels also significantly contributed to group discrimination. These findings allow to propose that obesity-associated disturbances in trace element and mineral status may at least partially contribute to metabolic risk in obese subjects.

## 1. Introduction

Obesity is considered as a worldwide epidemic affecting 11% men and 15% women worldwide [1]. Due to the role of obesity as the key component of metabolic syndrome, it accounts for 2.8 million deaths worldwide [2].

Micronutrient deficiency is considered as one of the characteristic patterns in obesity due to increased consumption of refined foods [3]. Correspondingly, multiple studies demonstrated impaired iron [4], magnesium [5], and zinc [6] in obese subjects. However, data on the association of obesity and selenium are contradictory and/or insufficient [7]. In contrast, accumulating evidence demonstrate that obesity may be associated with copper accumulation [8]. However, multiple contradictions regarding the association between obesity and trace element status exist [9,10]. A significant proportion of contradictory results may be attributable to the use of various substrates and markers for assessment of trace element and mineral status.

A number of studies have been aimed at the assessment of the relationship between trace elements and minerals in obesity and metabolic risk. Particularly, it has been demonstrated that certain patterns of hair trace element content may be characteristic for metabolically healthy obesity [11]. However, using urine for assessment of trace element status resulted in a distinct outcome [12]. We have also demonstrated that serum, hair, and dietary trace element levels are differentially associated with metabolic parameters in obese subjects [13]. However, the association between altered metabolic profile and mineral status is still contradictory.

Therefore, the objective of the study was to evaluate serum, hair, and urinary trace element and mineral content in normal-weight and obese women in relation to metabolic risk factors, as well as to estimate the contribution of trace elements in discrimination between the groups.

## 2. Materials and Methods

### 2.1. Ethical Statement

The study protocol has been approved by Ethics Committee, Poznan University of Medical Sciences (approval no. 1104/16 and no. 450/17) and fulfilled the requirements of The Declaration of Helsinki (1975 revision with amendments). The study is a part of a research which has been registered on ClinicalTrials.gov NCT03439540.

### 2.2. Study Design

Informed consent in writing has been obtained from each patient. Obesity was diagnosed on the basis of body mass index according to WHO criteria (BMI ≥ 30 kg/m^2^). Patients were recruited to two groups: study group with obesity and control group with normal-weight. A total of 80 women aged 30–70 years-old (y.o.) were enrolled in the obese group (n = 40) and normal-weight group (n = 40).

Patients who met all of the following inclusion criteria were enrolled: written informed consent; women aged 18 years and more; adequate value of BMI index (≥30 kg/m^2^ to the study group and 18.5–24.9 kg/m^2^ to the control group), stable body weight one month prior to enrolment (acceptable deviation was ±1 kg). The exclusion criteria were the following: diabetes, secondary form of obesity, diseases of the gastrointestinal tract, hepatic cirrhosis, dyslipidemia, hypertension requiring the implementation or modification of pharmacological treatment in the last 3 months before the enrollment or during the intervention, use of any dietary supplements within the last month before enrolment or during the study, a clinically relevant acute or chronic inflammatory process in the respiratory, digestive or genitourinary tract or in the mouth, throat, paranasal sinuses or connective tissue disease, arthritis, smoking, alcohol abuse, drug use, pregnancy, lactation, hormone replacement therapy. Patients’ age and sex were self-reported. Patients who met all inclusion criteria and did not present any of the exclusion criteria were included into the study. The occurrence of any of exclusion criterion during the study resulted in withdrawal of the patient from the trial.

### 2.3. Bioimpedance

Body composition analysis was determined using electrical bioimpedance InBody 370 device (InBody Bldg, Seoul, Korea), percentile fat mass (FAT%) was performed.

### 2.4. Anthropometric Measurements

Anthropometric parameters and body composition analyses were performed in the conditions of metabolic laboratory in patients wearing light clothes without shoes, fasting, at morning, after night-long sleep. BMI was calculated as the body mass divided by the height squared (kg/m^2^). The waist circumference (WC) was measured to the nearest 0.5 cm in the horizontal plane midway between the lowest rib and the iliac crest at the end of normal expiration.

### 2.5. Blood Pressure Measurement

Blood pressure measurements were performed using digital electronic tensiometer (model 705IT TM, Omron Corporation, Kyoto, Japan). Standard or large-size cuffs for adults were used. During the measurement patient was sitting in a chair for >5 min, feet on floor, back supported, after relaxation, with empty bladder. Three subsequent measurements were performed, and the mean was calculated. Heart rate (HR) was measured in the same conditions.

### 2.6. Blood, Urine, and Hair Sampling

Blood samples have been collected after all-night sleep, at the morning, fasting, at room temperature, without previous caffeine consumption. Before collection patient was lying supine in silence for 30 min. Blood samples were collected from ulnar vein into serum separation tubes. After preparation serum samples have been frozen and secured in −80 °C.

Urine samples were collected in the morning after overnight fasting using commercially-available plastic Vacuette^®^ Urine Collection Cups. Only the second portion of the first morning urine was collected and subsequently stored frozen at −30 °C.

Hair samples were collected at the day of blood and urine sampling. Proximal parts (1–2 cm, total weight of 0.1 g) of occipital scalp hair strands were collected after washing hair using ethanol-precleaned stainless steel scissors. Hair samples were stored in a laboratory at room temperature until analysis.

### 2.7. Biochemical Analysis

Serum levels of lipids–including total cholesterol (TCH), high-density lipoprotein cholesterol (HDL), and triglycerides (TG) were assayed by routine enzymatic methods with a Dimension EXL with LM Integrated Chemistry System Analyzer. Low-density lipoprotein cholesterol (LDL) was calculated from Friedewald’s formula. Glucose concentration was assessed by an enzymatic method with hexokinase, glucose HK gen.3 (GLUC.3), and insulin concentration was assessed by electrochemiluminescence immunoassay (Cobas analyzer). Homeostasis model assessment of insulin resistance (HOMA-R) was calculated based on fasting glucose concentration (mg/dL) and fasting insulin concentration (mU/mL) using the standard equation: (fasting plasma glucose/fasting plasma insulin)/405.

The accuracy and precision of the techniques used to assess all parameters were validated. Accuracy was assessed by means of the recovery value, which ranged between 95% and 109%. The variability coefficient did not exceed 10%.

### 2.8. Hair, Serum, and Urine Sample Preparation

The collected hair samples were washed with acetone and 18.2 MΩ · cm deionized water (Labconco Corp., Kansas City, MO, USA) with subsequent drying on air to a stable weight. Washed hair samples were subjected to microwave-assisted acid digestion in Berghof SpeedWave-4 DAP-40 system (Berghof Products + Instruments GmbH, 72800 Eningen, Germany) at microwave frequency, 2.46 GHz; power, 1450 W. Briefly, 50 mg dry hair samples were digested in Teflon tubes with 5 mL of concentrated (65%) nitric acid (Sigma-Aldrich Co., St. Louis, MO, USA) for 20 min at 170–180 °C. After cooling the system, the samples were adjusted to a final volume of 15 mL with distilled deionized water.

In turn, serum and urine samples were diluted 1:15 with an acidified (pH = 2.0) diluent containing (*v*/*v*) 1-Butanol 1% (Merck KGaA, Darmstadt, Germany), Triton X-100 0.1% (Sigma-Aldrich, Co., St. Louis, MO USA), and HNO_3_ 0.07% (Sigma-Aldrich, Co., St. Louis, MO, USA) in distilled deionized water.

### 2.9. ICP-MS Analysis

The analysis of calcium (Ca), copper (Cu), iron (Fe), magnesium (Mg), selenium (Se), vanadium (V), and zinc (Zn) levels in the studied samples was performed using inductively-coupled plasma mass-spectrometry (ICP-MS) at NexION 300D (PerkinElmer Inc., Shelton, CT, USA) equipped with a 7-port FAST valve and ESI SC-2 DX4 autosampler (Elemental Scientific Inc., Omaha, NE, USA). External calibration of the ICP-MS system was performed with 0.5, 5, 10 and 50 μg/L solutions of the studied elements. Calibration solutions were prepared from the Universal Data Acquisition Standards Kit (PerkinElmer Inc., Shelton, CT, USA). Internal on-line standardization was performed using 10 μg/L solutions of yttrium-89 and rhodium-103. The solutions were prepared from commercially available Yttrium (Y) and Rhodium (Rh) Pure Single-Element Standard (PerkinElmer Inc. Shelton, CT, USA) on a matrix containing 1-butanol 8% (Merck KGaA, Gernsheim, Germany), Triton X-100 0.8% (Sigma-Aldrich Co., St. Louis, MO, USA), tetramethylammonium hydroxide 0.02% (Alfa Aesar, Ward Hill, MA, USA) and ethylenediaminetetraacetic acid 0.02% (Sigma-Aldrich Co., St. Louis, MO, USA). The results of serum analysis were expressed as µg/mL for all elements except V (ng/mL). Data on hair and urinary trace element and mineral content are provided for all elements as µg/g and µg/mL, respectively.

### 2.10. Laboratory Quality Control

Laboratory quality control was performed using the reference materials of human hair (GBW09101, Shanghai Institute of Nuclear Research, Shanghai, China), plasma (ClinChek^®^ Plasma Control, Levels I, II, Lot 1286), and urine (ClinChek^®^ Urine Control, Levels I, II, Lot 1227). The recovery rates for reference materials of human hair, plasma, and urine were 91–107%, 93–105%, and 91–108%, respectively. In addition, sampling procedure purity was evaluated using blank samples of 18.2 MΩ · cm deionized water (Labconco Corp., Kansas City, MO, USA) that underwent all steps of sampling procedures and storage.

### 2.11. Statistical Analysis

The obtained data were processed Statistica 10.0 (Statsoft, OK, USA). Data distribution was evaluated using the Shapiro–Wilk test. Trace element and mineral levels were characterized by skewed distribution, thus being expressed as median and the respective interquartile range (IQR) boundaries. In turn, metabolic parameters followed Gaussian distribution and descriptive statistics included mean and the respective standard deviation. Group comparisons were performed using analysis of covariance (ANCOVA) adjusted for age, BMI, and metabolic parameters using Bonferroni post-hoc analysis. Data on trace element and mineral levels characterized by skewed distribution were subjected to log-transformation prior analysis. Correlation analysis was performed using Spearman rank correlation coefficient. Multiple linear regression analysis was performed in order to reveal independent association between BMI as a dependent variable and trace element and mineral levels in various biosamples as independent predictors with adjustment for metabolic parameters (routine predictors). Model 1 incorporated only metabolic parameters, blood pressure, and age as independent variables, whereas Models 2, 3, and 4 also included data on serum, hair, and urinary trace element and mineral levels, respectively. Discriminant analysis was performed in order to assess significant discrimination between the study groups based on metabolic parameters and trace element content in biosamples with evaluation of significance of the distance between group vectors. The level of significance was set as *p* < 0.05 for all statistical methods.

## 3. Results

The obtained data demonstrate that obese subjects were characterized by 63% and 60% higher body weight and BMI values as compared to lean controls, respectively (Table 1). At the same time, no significant differences in age and height were revealed. SBP and DBP in obese examinees exceeded the control values by 11% and 13%, whereas heart rate did not differ significantly between the groups.

Obesity was associated with alteration of metabolic profile in the studied group of subjects (Table 1). Surprisingly, serum total cholesterol levels were 14% lower in obese patients when compared to the control subjects. At the same time, serum HDL-C levels were found to be 41% lower whereas LDL-C concentration tended to increase in obesity. Moreover, serum TG levels in the examined obese subjects were 30% higher than those in control examinees, altogether being indicative of atherogenic lipid profile.

The analysis of serum glucose concentration failed to reveal any group difference in serum glucose in the studied sample of obese patients, whereas serum insulin levels were found to be 40% higher. Correspondingly, 48% higher HOMA-IR values in obese examinees are indicative of insulin resistance without manifest hyperglycemia.

Obesity was associated with altered trace elements in serum, hair, and urine (Table 2). Serum Ca, Fe, Mg, Se, V, and Zn levels were found to be 28%, 27%, 14%, 37%, 42%, and 8% lower in obese subjects as compared to lean controls. At the same time, serum Cu levels in obese examinees significantly exceeded the respective control values by 3%.

Hair analysis revealed 35% and 41% lower hair Fe and Mg content in obese examinees as compared to the control values, respectively. Hair V levels were found to be twofold lower than those in the lean controls. In turn, the observed 15% and 19% decrease in hair Se and Zn content in the obese group, these differences were only nearly significant (*p* < 0.1).

Urinary trace element and mineral levels were also affected in obesity. Particularly, urinary Se and V concentrations were 31% and 41% lower when compared to normal-weight controls, respectively. In turn, urinary Fe levels in obese patients were more than two-fold higher than those in lean controls.

Correlation analysis demonstrated that trace element and mineral levels in the studied samples were significantly associated with anthropometric and metabolic parameters (Table 3). Particularly, BMI values were characterized by a significant inverse correlation with serum Ca, Fe, Mg, V, Zn levels, hair Mg and V content, as well as urinary Se concentrations. Systolic blood pressure was negatively associated with serum Ca, Mg, Se, V, and Zn levels, as well as hair Mg content. DBP inversely correlated with serum Ca, Mg, Se, V, and Zn concentrations, whereas serum Cu level was positively associated with this parameter. Serum HDL-C levels positively correlated with serum Ca, Fe, Mg, Se, V, Zn, hair V, and urinary Se concentration. Similar, although less pronounced associations were revealed in the case of serum TC. In turn, serum Ca, Mg, Se, V, and Zn were inversely associated with TG levels. Certain associations were revealed between trace element and mineral levels and markers of carbohydrate metabolism. In turn, circulating insulin levels were characterized by inverse association with serum Ca, Fe, Mg, Se, Zn, as well as urinary Mg and Se concentration. At the same time, serum Cu level positively correlated with insulin levels. Correspondingly, serum Cu concentrations directly correlated with HOMA-IR values, whereas serum Mg and Zn, as well as urinary Mg were inversely associated with the latter. Heart rate and fasting plasma glucose were slightly associated with trace element and mineral profile. Specifically, heart rate was characterized by weak inverse correlation with serum Zn (r = −0.203) and urinary Se (r = −0.188) levels, whereas serum glucose negatively correlated only with hair Se content (r = −0.185).

In view of the observed correlation between mineral levels, metabolic and anthropometric parameters, multiple linear regression analysis was performed in order to reveal the independent association between trace element and mineral levels and BMI values after adjustment for metabolic markers (Table 4). Model 1 incorporating only metabolic parameters and blood pressure values accounted for 53% variability of BMI. HDL-C and SBP values were considered as negative and positive predictors of BMI. Including serum trace element and mineral levels into the model (Model 2) significantly increased predictive value of the model by more than 18% (from 53.0% to 71.8%). Serum Ca, Se, and Zn levels were found to be inversely associated with BMI values, whereas serum Cu concentration was directly associated with higher BMI. Models 3 and 4 incorporating hair and urinary trace element and mineral levels did not improve predictive ability of the baseline model (Model 1). None of hair or urinary element levels were found to be significantly associated with BMI.

Given the tight independent association between serum trace element and mineral levels with BMI, the most effective model (Model 2) was also adjusted for percentage body fat assessed by bioimpedance analysis. The obtained data demonstrate that the resulting model accounts for nearly 80% of BMI variability (Multiple R = 0.909; Multiple R^2^ = 0.827; Adjusted R^2^ = 0.799; *p* for a model < 0.001). It is clear that body fat percentage was considered as the most significant predictor for BMI (β = 0.455; *p* < 0.001). Serum Cu (β = 0.110; *p* = 0.016), Se (β = −0.188; *p* = 0.009), and Zn (β = −0.118; *p* = 0.018) levels remained significantly associated with BMI even after adjustment for body composition. In addition, serum TG levels (β = 0.107; *p* = 0.044) and SBP (β = 0.149; *p* = 0.014) values were found to be directly associated with BMI values.

**Figure 1:**
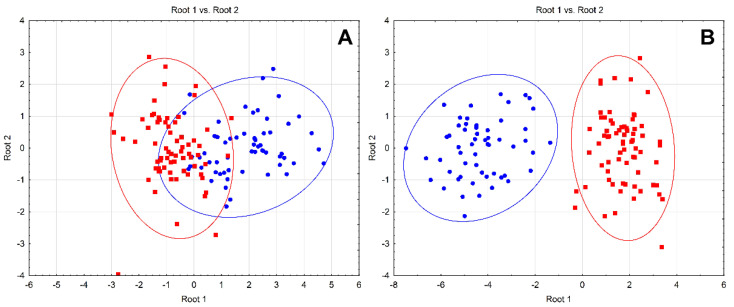
Discriminant analysis score plot of normal body weight (blue) and obesity (red) based on the patterns of metabolic parameters and blood pressure (**A**) or serum trace elements, metabolic parameters, and blood pressure (**B**).

Discriminant analysis (Figure 1) was performed in order to reveal the contribution of the particular variables into discrimination between the groups. Although the model incorporating metabolic parameters and blood pressure values revealed significant distance between the groups (MD^2^ = 7.20; *p* < 0.001), complete discrimination was not observed (Figure 1). TC, HDL-C, LDL-C (all *p* < 0.001), SBP (*p* = 0.004), and circulating insulin (*p* = 0.041) values were found to contribute significantly into the model. Inclusion of serum trace element and mineral levels, characterized by the most significant association with BMI, significantly increased the distance between the groups (MD^2^ = 36.78; *p* < 0.001) resulting in complete discrimination. Serum Ca, V, Zn (all *p* < 0.001), Cu (*p* = 0.002), Se (*p* = 0.011), and Mg (*p* = 0.036) levels contributed significantly to group discrimination along with HDL-C (*p* < 0.001) and SBP (*p* = 0.031) values.

## 4. Discussion

The obtained data clearly demonstrate that obese patients are characterized by altered trace element and mineral levels in the studied biosamples.

The obtained data clearly demonstrate altered iron status in obese examinees that may occur due to chronic inflammatory response in obesity with subsequent hepcidin overproduction [14]. The results of meta-analysis involving data from 13,393 overweight/obese and 26,621 normal weight subjects revealed significantly lower serum Fe levels in obesity [4]. At the same time, urinary iron excretion was found to be more than twofold higher in obese subjects when compared to lean controls, being indicative of obesity-associated oxidative stress [15]. Altered serum Fe levels were also inversely associated with circulating insulin levels as earlier demonstrated [3].

The observed increase in serum Cu levels in obese subjects corroborates the findings of the most recent meta-analysis demonstrating a positive association between increased serum Cu and obesity [8]. Moreover, a detailed study demonstrated that increased serum and adipose tissue Cu and cuproprotein levels are associated with leptin and insulin levels [16]. These findings are generally in agreement with the results of our previous experimental study demonstrating the potentiating effect of Cu intake on diet-induced obesity and adipose tissue hypertrophy [17]. Further analysis demonstrated tight association between high Cu levels and metabolic risk markers being in agreement with the observation of direct relationship between serum Cu and insulin resistance in prediabetic and diabetic postmenopausal women [18]. Positive association between Cu status and atherogenic lipid profile may underlie increased risk of atherosclerosis [19] and be mediated through the impact of Cu on lipid metabolism [20]. High serum Cu levels were also found to be associated with increased blood pressure [21], although the existing data are rather contradictory [22].

Se levels in the studied biosamples were found to be inversely associated with obesity that may occur due to the role of selenium and adipose tissue-resident selenoproteins in adipocyte physiology when both increased and decreased selenoprotein expression may result in adipocyte dysfunction [17]. The observed serum Se levels in obese subjects were also far lower than the reference ranges of 90–100 µg/l required for normal selenoprotein function [23]. Similar association was observed between Se status and diabetes [24] arising from the role of Se-dependent redox regulation of insulin signal transduction [25]. At the same time, hair Se levels were found to be inversely associated with HOMA-IR values in obese subjects [26]. The findings of increased endothelial dysfunction, vascular oxidative stress, and impaired NO-mediated vascular reactivity [27] may underlie the observed inverse association between Se status and blood pressure.

Zinc status was found to be inversely associated with anthropometric parameters including body composition and metabolic risk factors. This observation corresponds to the earlier studies summarized in a recent meta-analysis demonstrating low serum Zn levels in obese children and adults [6]. Our previous study also revealed a significant reduction in serum, hair, and urinary Zn levels in obesity [28]. Earlier, we also demonstrated a significant inverse association between serum Zn, insulin, and total cholesterol levels [29], altogether corresponding to the role of Zn and Zn-containing metalloproteins like Zn-α2-glycoprotein in adipose tissue functioning, insulin signaling, and lipid metabolism [30]. Correspondingly, Zn supplementation was shown to improve body weight, insulin signaling, and systemic inflammation in obese individuals [31]. Inverse association between Zn status and blood pressure may be at least partially attributable to the newly demonstrated role of Zn deficiency in increasing renal sodium reabsorption [32].

Vanadium is known to play a significant role in insulin signaling though negative modulation of PTP1B and PTEN activity and stimulating downstream insulin signaling [33]. At the same time, our previous experimental [34] and human [28] studies demonstrated an inverse association between V levels and obesity. Correspondingly, V supplementation was shown to modulate adipogenesis [35], thus being indicative of its potential antiobesity effects [36].

The obtained data are generally in agreement with the previous studies demonstrating an association between dietary Ca deficiency and obesity [37]. Correspondingly, Ca supplementation was shown to reduce body weight as demonstrated by meta-analysis including data from 4733 participants [38]. The protective role of adequate calcium supply in obesity may be mediated by its impact on adipogenesis, thermogenesis, lipid metabolism, and gut microbiota [39]. Moreover, calcium may up-regulate the insulin signaling pathway, thus reducing insulin resistance in obesity [40].

The results of meta-analysis also demonstrate high rate of Mg deficiency in patients with obesity [41]. Mg deficiency was also found to be associated with insulin resistance in obesity [42] due to modulation of insulin receptor phosphorylation as well as insulin secretion [43]. Particularly, increased dietary Mg intake was associated with lower BMI and glucose levels [44]. Serum Mg levels is tightly associated with metabolic risk factors in obesity [45]. The role of Mg in modulating blood pressure may be mediated by the impact of Mg on cardiac output [46].

However, the revealed associations were sample-dependent, being observed predominantly in serum. Although findings on hair and urinary mineral content are generally in line with the patterns observed in serum, only serum trace element and mineral levels significantly correlated with metabolic parameters, being in agreement with the role of serum as the most reliable marker for trace element status assessment [47]. Mechanistically, more tight association between serum elements and metabolic parameters also assessed in serum may be at least partially mediated by the same matrix effects including dilution. In addition, the studied parameters in serum are subjected to homeostatic regulation, whereas urine and especially hair mineral levels are more variable. Our previous study also demonstrated a tight interrelation between serum Zn, Cr, and V levels and BMI, whereas in hair, only Se content was independently associated with BMI [28]. Serum mineral levels were also associated with more metabolic parameters (TG, TC, and HDL) than hair content (HDL, TG) [13]. Błażewicz et al. (2013) also observed more correlations between plasma/whole blood metal levels and BMI in children than in the case of urine [9].

This study has some limitations which have to be pointed out. The greatest limitation of the study is the relatively small number of patients in both groups. This was mainly due to rigorous inclusion and exclusion criteria, which allowed us to include a homogenous population of women not encumbered by states which could have affected the quality of the results. It is also notable that urinary trace element levels were not adjusted for creatinine, which could significantly reduce the variability of metal excretion data. Furthermore, in the present study, we did not determine nutritional factors and environmental factors that may affect the concentrations of elements in the analyzed samples.

## 5. Conclusions

The obtained data demonstrate that reduced Ca, Fe, Mg, Se, V, and Zn, as well as increased Cu levels are independently associated with obesity, being also characterized by a significant relationship with insulin resistance, atherogenic lipid profile, and increased blood pressure. These findings allow to propose that obesity-associated disturbances in trace element and mineral status may at least partially contribute to metabolic risk in obese subjects. However, longitudinal studies are required to clarify the causal relationship between altered mineral status and metabolic risk on obesity.

## Figures and Tables

**Table 1:** Anthropometric and metabolic parameters in subjects with normal weight and obesity.

Parameter	Control (n = 40)	Obese (n = 40)	*p* Value
Age, y.o.	52.5 ± 11.8	51.4 ± 12.2	0.603
Height, cm	162.2 ± 6.6	163.6 ± 6.9	0.209
Body mass, kg	59.9 ± 6.2	97.4 ± 16.8	<0.001 *
BMI, kg/m^2^	22.7 ± 1.4	36.4 ± 5	<0.001 *
BF, %	30.4 ±5.4	46.3 ±5.4	<0.001 *
SBP, mmHg	128.4 ± 18.5	142.8 ± 18.6	<0.001 *
DBP, mmHg	76.3 ± 11.5	85.9 ± 10.7	<0.001 *
HR, bpm	77.7 ± 13.8	80.2 ± 11.9	0.381
TC, mg/dL	213.3 ± 43.4	184 ± 44.2	<0.001 *
HDL-C, mg/dL	77.7 ± 18.5	46 ± 17.7	<0.001 *
LDL-C, mg/dL	115.2 ± 40.6	120.8 ± 45.6	0.379
TG, mg/dL	110.3 ± 54.3	143 ± 69.3	0.003 *
FPG, mg/dL	90.3 ± 14.9	88.4 ± 25.9	0.622
Insulin, µU/mL	11.4 ± 8.5	16 ± 11.4	0.012 *
HOMA-IR	2.5 ± 2	3.7 ± 3.4	0.023 *

Data expressed as Mean ± SD; *—significant group difference at *p* < 0.05 according to ANCOVA with Bonferroni adjustment; BMI—body mass index, BF—body fat percentage, SBP—systolic blood pressure, DBP—diastolic blood pressure, HR—heart rate, TC—total cholesterol, HDL-C—high density lipoprotein cholesterol, LDL-C—low density lipoprotein cholesterol, TG—triglycerides, HOMA-IR—Homeostatic Measurement Assessment-Insulin Resistance.

**Table 2:** Serum, hair, and urinary trace element and mineral levels in normal weight and obese subjects.

Element	Control (n = 40)	Obese (n = 40)	*p* Value
Serum			
Ca, µg/mL	101.8 (97.9–106.6)	74.2 (70.7–78.6)	<0.001 *
Cu, µg/mL	1.136 (0.938–1.315)	1.170 (1.07–1.290)	0.018 *
Fe, µg/mL	1.243 (0.923–1.505)	0.910 (0.736–1.150)	0.001 *
Mg, µg/mL	22.7 (21.3–23.3)	19.5 (17.9–20.7)	<0.001 *
Se, µg/mL	0.106 (0.096–0.116)	0.067 (0.061–0.078)	<0.001 *
V, ng/mL	5.316 (4.412–5.766)	3.1 (2.7–3.2)	<0.001 *
Zn, µg/mL	0.88 (0.805–0.947)	0.814 (0.762–0.87)	0.001 *
Hair			
Ca, µg/g	1406.3 (773.6–2755.9)	1738 (650–2929)	0.699
Cu, µg/g	14.456 (10.705–25.87)	13.5 (12.03–18.57)	0.936
Fe, µg/g	14.381 (9.183–24.417)	9.3 (6.75–13.58)	0.008 *
Mg, µg/g	152.3 (67.3–265.5)	89.7 (53.4–154)	0.017 *
Se, µg/g	0.391 (0.331–0.461)	0.332 (0.278–0.42)	0.074
V, µg/g	0.014 (0.01–0.034)	0.007 (0.004–0.011)	<0.001 *
Zn, µg/g	198.7 (160.3–227.5)	160 (132–188)	0.062
Urine			
Ca, µg/mL	97.7 (53.5–154.5)	96.2 (46.3–157)	0.932
Cu, µg/mL	0.011 (0.008–0.017)	0.011 (0.008–0.016)	0.808
Fe, µg/mL	0.022 (0.015–0.041)	0.046 (0.018–0.213)	0.012 *
Mg, µg/mL	77.7 (50.7–128.8)	67.7 (47.7–98.3)	0.142
Se, µg/mL	0.026 (0.015–0.041)	0.018 (0.01–0.026)	0.016 *
V, ng/mL	0.068 (0.041–0.157)	0.040 (0.005–0.09)	0.006 *
Zn, µg/mL	0.284 (0.183–0.465)	0.282 (0.177–0.431)	0.400

Data expressed as Median and the respective IQR boundaries; *—significant group difference at *p* < 0.05 according to ANCOVA with Bonferroni adjustment.

**Table 3:** Correlation between trace element and mineral levels in the studied biosamples and BMI, blood pressure, and metabolic parameters in the studied cohort.

Element	BMI, kg/m^2^	BF, %	SBP, mmHg	DBP, mmHg	TC, mg/dL	HDL-C, mg/dL	TG, mg/dL	insulin, µU/mL	HOMA-IR
Serum Ca	−0.776 *	−0.748*	−0.343 *	−0.369 *	0.339 *	0.605 *	−0.245 *	−0.174 *	−0.151
Serum Cu	0.293 *	0.245*	0.156	0.195 *	−0.045	−0.153	0.097	0.364 *	0.302 *
Serum Fe	−0.193 *	−0.260*	−0.167	−0.138	0.141	0.191 *	−0.027	−0.174 *	−0.143
Serum Mg	−0.592 *	−0.560*	−0.321 *	−0.323 *	0.205 *	0.477 *	−0.269 *	−0.214 *	−0.186 *
Serum Se	−0.686 *	−0.610*	−0.183 *	−0.248 *	0.376 *	0.569 *	−0.210 *	−0.181 *	−0.146
Serum V	−0.544 *	−0.553*	−0.219 *	−0.264 *	0.272 *	0.445 *	−0.177 *	−0.097	−0.089
Serum Zn	−0.261 *	−0.218*	−0.250 *	−0.206 *	0.250 *	0.222 *	−0.219 *	−0.274 *	−0.224 *
Hair Mg	−0.228 *	−0.204*	−0.208 *	−0.151	0.075	0.160	−0.115	−0.098	−0.084
Hair Se	−0.130	−0.103	−0.031	−0.022	0.141	0.015	0.109	−0.019	−0.058
Hair V	−0.235 *	−0.194*	−0.027	−0.065	0.059	0.204 *	−0.084	0.166	0.146
Urine Mg	−0.132	−0.120	−0.144	−0.142	0.080	0.157	−0.062	−0.212 *	−0.215 *
Urine Se	−0.239 *	−0.274*	−0.106	−0.126	0.223 *	0.239 *	0.030	−0.185 *	−0.157

Data expressed as Spearman rank correlation coefficient (r); *—significant correlation at *p* < 0.05; BMI—body mass index, SBP—systolic blood pressure, DBP—diastolic blood pressure, HR—heart rate, TC—total cholesterol, HDL-C—high density lipoprotein cholesterol, LDL-C—low density lipoprotein cholesterol, TG—triglycerides, HOMA-IR—Homeostatic Measurement Assessment-Insulin Resistance.

**Table 4:** Multiple regression analysis of the association between trace element and mineral levels with BMI after adjustment for multiple clinical and laboratory markers.

Parameter	Model 1		Model 2 (Serum)		Model 3 (Hair)		Model 4 (Urine)	
	β	*p*	β	*p*	β	*p*	β	*p*
FPG, mg/dL	−0.026	0.824	−0.009	0.924	−0.090	0.473	0.003	0.984
TC, mg/dL	−0.149	0.090	0.012	0.869	−0.132	0.163	−0.090	0.376
HDL-C mg/dL	−0.529	<0.001 *	−0.164	0.029 *	−0.476	<0.001 *	−0.540	<0.001 *
LDL-C, mg/dL	0.040	0.631	−0.052	0.446	0.040	0.658	0.053	0.562
HOMA-IR	0.323	0.279	0.097	0.692	0.384	0.217	0.348	0.274
TG, mg/dL	0.071	0.380	0.096	0.139	0.087	0.290	0.047	0.613
Insulin, µU/mL	−0.222	0.411	0.003	0.989	−0.241	0.395	−0.242	0.406
Age, y.o.	−0.033	0.638	0.011	0.841	−0.011	0.884	−0.032	0.688
SBP, mmHg	0.212	0.024 *	0.155	0.041 *	0.170	0.083	0.250	0.016 *
DBP, mmHg	0.159	0.085	0.021	0.773	0.159	0.090	0.135	0.186
HR, bpm	0.014	0.849	0.055	0.338	0.016	0.830	0.019	0.818
Ca, µg/mL	-	-	−0.391	<0.001 *	−0.001	0.996	0.015	0.880
Cu, µg/mL	-	-	0.179	0.001 *	0.011	0.867	0.035	0.689
Fe, µg/mL	-	-	0.037	0.481	−0.062	0.357	0.030	0.701
Mg, µg/mL	-	-	−0.021	0.777	−0.093	0.296	−0.211	0.083
Se, µg/mL	-	-	−0.230	0.009 *	−0.127	0.075	0.065	0.412
V, ng/mL	-	-	−0.103	0.129	−0.111	0.091	0.168	0.147
Zn, µg/mL	-	-	−0.198	0.001 *	−0.002	0.974	0.035	0.755
Multiple R	0.755		0.871		0.777		0.761	
Multiple R^2^	0.570		0.759		0.603		0.579	
Adjusted R^2^	0.530		0.718		0.536		0.500	
*p* for a model	<0.001 *		<0.001 *		<0.001 *		<0.001 *	

Data expressed as regression coefficient (β) and the respective *p* values; *—association is significant at *p* < 0.05; BMI—body mass index, SBP—systolic blood pressure, DBP—diastolic blood pressure, HR—heart rate, TC—total cholesterol, HDL-C—high density lipoprotein cholesterol, LDL-C—low density lipoprotein cholesterol, TG—triglycerides, HOMA-IR—Homeostatic Measurement Assessment-Insulin Resistance.
